# Do difficulties in emotion regulation impact self-esteem and adult attachment? – the role of trauma

**DOI:** 10.1080/07853890.2021.1896150

**Published:** 2021-09-28

**Authors:** Ana V. Antunes, Carina Santos, Patrícia Oliveira, Telma C. Almeida

**Affiliations:** a Instituto Universitário Egas Moniz (IUEM), Egas Moniz Cooperativa de Ensino Superior, Caparica, Portugal; b Laboratório de Psicologia Egas Moniz (LabPSI), Centro de Investigação Interdisciplinar Egas Moniz (CiiEM), Egas Moniz Cooperativa de Ensino Superior, Caparica, Portugal

## Abstract

**Introduction:**

Experiencing trauma in childhood, adolescence or adulthood has severe negative impact on several areas of individuals' lives. Concerning their psychological health, consequences often arise in terms of emotional deficits, self-esteem and attachment [1]. Strategies of emotional regulation (ER) are extremely important in the individual’s development and have implications in their self-concept and self-esteem [2]. The literature suggests that a better ER is directly associated with better self-esteem [3]. Some studies also show an association between attachment and the level of self-esteem [4]. Better attachment in adults is associated with high and stable levels of self-esteem [5]. The objective of this research is to study the impact of ER, self-esteem and attachment in Portuguese adults (above 18 years old) who have experienced trauma. This study contributes to scientific innovation in this field, considering that we found results never obtained in a Portuguese sample.

**Materials and Methods:**

This study comprised 137 Portuguese individuals’ (69.3% women and 30.7% men) with ages between 18 and 70 years (*M* = 39.49, SD = 12.49), of which 74 (54.0%) experienced trauma in the last three years. This study was carried out by filling out online questionnaires and the link to the study was disclosed by e-mail and in social networks. The participants responded to a sociodemographic questionnaire, a checklist of some types of trauma experienced in the last three years, the Difficulties in Emotional Regulation Scale (DERS) [6], the Rosenberg Self-Esteem Scale (RSES) [7], and the Adult Attachment Scale (AAS-R) [8]. The study was conducted in accordance with all the ethical principles.

**Results:**

The results showed a significant negative correlation between the DERS and the total score of the RSES (*r* = −0.509, *p* < .001) as well as between the DERS Strategies subscale and the RSES (*r* = −0.541, *p* < .001). There was a significant statistical negative correlation between the DERS Awareness and the AAS-R (*r* = −0.232, *p* = .006), and a significant statistical positive correlation between the total score of the DERS (*r* = 0.557, *p* < .001), the DERS Non-Acceptance (*r* = 0.500, *p* < .001) the DERS Strategies (*r* = 0.516, *p* < .001) and the AAS-R Anxiety. The results also showed significant statistical differences between individuals who experienced trauma in the Anxiety dimension of the AAS-R [*F*(1,136) = 8.91, *p* = .003]. Those who experienced trauma showed higher anxiety (*M* = 2.19, SD = 0.75).

**Discussion and conclusions:**

The results showed that if difficulties of emotional regulation and limited access to emotional regulation strategies decreases, self-esteem increases, which corroborates the literature [3]. Concerning the adult attachment, we found that if anxiety increases, difficulties of emotional regulation also increase, as well as lack of acceptance of emotional responses and limited access to emotional regulation strategies. Some studies reveal that attachment styles influence the strategies to express and regulate emotions [9]. All these findings have an impact on clinical and social levels, as they guide therapists to work in these specific areas with individuals who have suffered some form of trauma. Thus, our results help to develop psychological intervention programs to prevent psychopathology. Further studies should focus on the results of preventive and interventive efficacy of therapies implemented in society that focus on emotion regulation, self-esteem, and adult attachment in traumatised individuals.
